# Hydrogels with reversible stiffness modulation: new materials for studying dynamic mechanical cues that regulate cell behavior

**DOI:** 10.3389/fbioe.2025.1710978

**Published:** 2025-12-05

**Authors:** Pinxue Li, Yubo Liu, Shuaiyi Liu, Meng Zhou, Xing Wang, Fangyuan Yu

**Affiliations:** 1 Department of Sports Medicine & Shoulder, Beijing Jishuitan Hospital, Capital Medical University, Beijing, China; 2 School of Medicine, Nankai University, Tianjin, China; 3 Senior Department of Orthopedics, Fourth Medical Center of People’s Liberation Army General Hospital, Beijing, China; 4 National Clinical Research Center for Orthopaedics, Sports Medicine & Rehabilitation, Beijing, China; 5 Beijing National Laboratory for Molecular Sciences, Institute of Chemistry, Chinese Academy of Sciences, Beijing, China; 6 University of Chinese Academy of Sciences, Beijing, China

**Keywords:** hydrogel, tunable stiffness, cell behavior, extracellular matrix, tissue engineering

## Abstract

As the fundamental environment for cell survival, the extracellular matrix (ECM) not only serves as the substrate for cell function and structure formation but also guides cell activities through its dynamic physical properties. Therefore, the relationships by which the physical and mechanical properties of the ECM guide cell behavior such as growth, development, differentiation and reproduction are important to understand. Because substrate stiffness is an important physical property that influences cell behavior, this paper focuses on the relationship between stiffness and cell behavior. Hydrogels, as networks of hydrophilic polymer chains, are an excellent model for the physical properties of the ECM in cellular studies due to its multiple similarities with the ECM. This review classifies hydrogels in terms of their origin and their relative stiffness and presents an overview of their formation, properties, regulation, and applications. We believe that hydrogels with variable elastic moduli will continue to be of considerable use in future studies to further elucidate the effects of mechanical cues on cell behavior.

## 1 Introduction

The extracellular matrix (ECM), a complex, dynamic, cross-linked meshwork with tethered biomolecules, is fundamental to the formation and function of tissues and organs. It offers crucial physical support for cells and generates essential biochemical and biomechanical signals that are required for tissue development (Xie et al., 2023). The best-studied physical properties of the ECM are its stiffness, porosity, insolubility, and topography. Matrix stiffness, which is described by the elastic modulus (Young’s modulus or storage modulus), is known to play a crucial role in cell adhesion, migration, proliferation and differentiation (Xie et al., 2023; Rosales and Anseth, 2016; Chaudhuri et al., 2020). Restructuring and remodeling of the tissue architecture occur through the degradation, deposition and modification of the ECM (Ting et al., 2021). Cells can sense mechanical cues (e.g., stiffness, contractility, surface pattern and dimensionality) that usually act in concert with other physicochemical and biochemical properties through cell membrane-bound receptors. Therefore, providing mechanical cues to guide cell behavior by altering the physical properties of the ECM is a promising approach.

Numerous studies have shown that matrix hardness can influence the direction of stem cell differentiation. Engler et al. cultured mesenchymal stem cells (MSCs) on polyacrylamide (PAAm) hydrogels with different degrees of cross-linking. PAAm hydrogels were fabricated to match the elastic modulus of the brain (0.1–l kPa), muscle (8–17 kPa), and collagenous bone tissue (25–40 kPa), and the degree of matrix stiffness promoted neurogenic, myogenic, and osteogenic MSC differentiation profiles (Engler et al., 2006), respectively. This pioneering work correlated mechanical cues with cellular behavior for the first time. The effect of matrix stiffness on cell fate has been increasingly studied, and matrix stiffness has been shown to be an important regulator of stem cell fate, especially in differentiation. Therefore, the mechanical properties of culture substrates for cellular studies need to be carefully designed.

Hydrogels are networks of hydrophilic polymer chains, sometimes called colloidal gels, in which water is the dispersion medium. The three-dimensional softness of the material is because the hydrophilic polymer chains are held together by cross-linking. Due to the intrinsic cross-linking, the structural integrity of the hydrogel network is not compromised by the addition of high concentrations of water (Warren et al., 2017). Because of their ability to tolerate a high water content and because they share various similarities with human tissues, hydrogels are ideal candidates for bioengineering applications. Hydrogels are good candidates for mimicking the physical properties of the ECM in cell studies. Mechanical cues from substrates play an important role in regulating cell behavior, and hydrogels have been widely used to mimic natural substrates for cell culture. With the rapid development of science and technology, various characterization techniques, including macroscopic and microscopic characterization, have been used to evaluate the dynamic mechanical properties of materials and tissues.

In this paper, we review and categorize the mechanisms used in the design of various hydrogels with variable stiffnesses. We first present relevant studies on the relationship between matrix stiffness and cell behavior, especially the effect of matrix stiffness on stem cell growth and differentiation. We then briefly describe how stem cells sense mechanical signals from the ECM and how they respond to these signals at the molecular level. In Section 3, we introduce hydrogels and describe the applications of various hydrogels in cellular research. Then, we summarize the unique applications of stiffness-variable hydrogels in bioengineering, including cell/organoid culture, tissue engineering, and immunomodulation. Finally, we present the prospects and challenges of using stiffness-variable hydrogels for studying cell behavior.

## 2 Modulation of cell behavior by stiffness

In 2004, a study by Engler et al. showed that substrate stiffness has a profound effect on the behaviors of MSCs (Engler et al., 2006). Culturing umbilical cord MSCs on polyacrylamide gels encapsulated with fibronectin of different stiffnesses (Young’s modulus 13–16, 35–38, 48–53, and 62–68 kPa) resulted in differences in their ability to adhere, proliferate, and spread. The maximum spreading of MSCs was observed at the highest matrix stiffness. Soft substrates promoted lipogenic cell differentiation with high expression of PPARγ and C/EBPα. The 48–53 kPa substrates induced MSC differentiation toward myoblasts with high expression of MOYG, whereas, MSCs cultured on hard substrates differentiated into osteoblasts with high expression of ALP, type I collagen, Runx2 and osteocalcin. In addition, bone marrow MSCs cultured on fibronectin-coated polyacrylamide hydrogels with varying stiffnesses (ranging from 13 to 68 kPa) exhibited increased adhesion, spreading, and proliferation with increasing matrix stiffness. At 62–68 kPa, the MSCs were polygonal with a wider distribution area and showed increased expression of Runx2, ALP, and bone bridging proteins.

**FIGURE 1:**
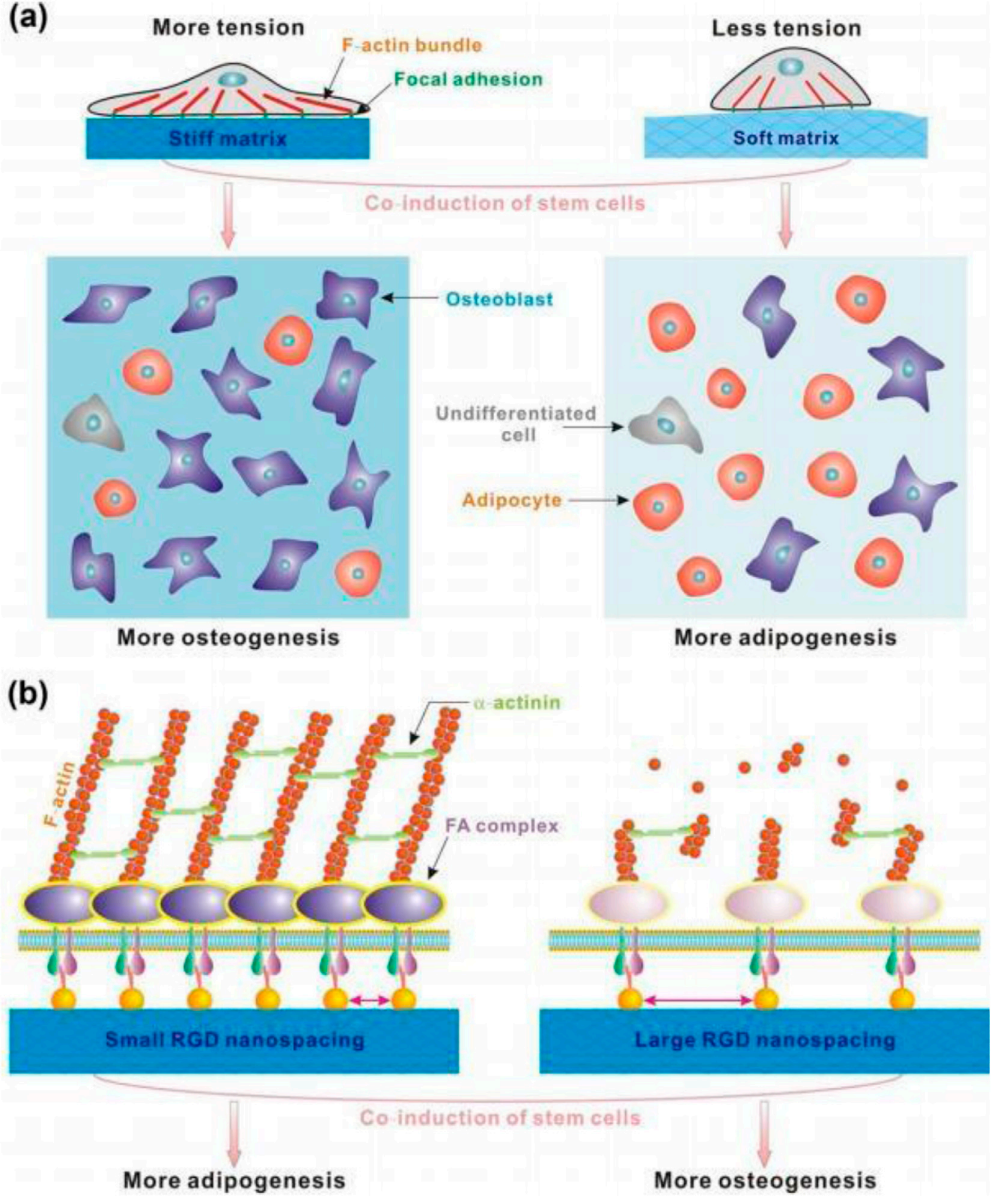
Schematic illustration of the effects of matrix stiffness and the organization of cell-adhesive ligands on stem cells. **(a)** Matrix stiffness effect. The strong mechanical feedback from a stiff hydrogel leads to increased activation of FA complexes and stronger cell tension. The corresponding inside-outside-in sensing leads to increased osteogenesis. **(b)** RGD nanospacing effect. Focal adhesions formed well on patterns with small nanospacing, but cells did not form cross-actin bundles above the critical adhesion nanospacing (ca. 70 nm). A large RGD nanospacing favors osteogenesis. The effect of RGD nanospacing implies the presence of an unknown outside-in signaling pathway. Reproduced with permission from Ye et al. (2015).

A follow-up study by Trappmann et al. (2012) examined human epidermal stem cells cultured on the surfaces of polydimethylsiloxane and PAAm hydrogel surfaces with stiffnesses between 0.1 kPa and 2.3 MPa, to which collagen was bound. The authors hypothesized that the density of cell adhesion proteins affects the cellular response more than the substrate stiffness itself does. The authors concluded that stem cells exert force on the ECM and that mechanical feedback is measured by the density of the anchors (Wen et al., 2014). Ye et al. (2015) studied polyethylene glycol (PEG) hydrogels and adherent arginine glycine-aspartic acid (RGD) peptides in an attempt to further isolate the effects of substrate stiffness and surface binding (Figure 1). They reported that matrix stiffness was a potent regulator of stem cell differentiation, and the RGD nanospacing affected the spreading area and differentiation of rat MSCs regardless of the hydrogel stiffness.

Stem cells perceive mechanical signals from the extracellular matrix (ECM) primarily through integrin-mediated adhesions, which link the ECM to the intracellular cytoskeleton. Upon binding ECM ligands, integrins cluster and recruit adaptor proteins such as talin and vinculin, enabling force transmission to actin filaments. Myosin II-driven contractility then generates tension within actin stress fibers, allowing cells to actively probe and respond to matrix stiffness. This mechanotransductive process, in which cells exploit the contractile properties of stress fibers to engage with their surrounding matrix, directly influences stem cell proliferation and differentiation (Even-Ram et al., 2006). Mechanical signaling to the cell regulates the differentiation of MSCs into mature, specialized cells through the activation of transcription factors, which upregulate genes responsible for the initiation and progression of differentiation in a particular cell lineage. The Hippo pathway is involved in MSC differentiation and is regulated by both intracellular and extracellular signals (Ma et al., 2019). The downstream effectors of the Hippo signaling pathway are yes-associated protein (YAP) and transcriptional coactivators with PDZ-binding motifs (TAZ) (Yamaguchi and Taouk, 2020). YAP and TAZ are essential signals for determining the fate of MSCs. The Hippo pathway is controlled through YAP/TAZ phosphorylation and nuclear translocation (Dobrokhotov et al., 2018). Furthermore, matrix stiffness can control the localization and activity of YAP/TAZ, which regulates cellular tension through structural and functional modulation of the cytoskeleton (Aragona et al., 2013). The stress exerted on MSCs is transmitted to the nucleus, which increases the nuclear membrane tension, resulting in nuclear pore dilatation and facilitating an abrupt nuclear influx of YAP (Elosegui-Artola et al., 2017). In MSCs cultured on rigid substrates (40 kPa) undergoing osteogenic differentiation, YAP/TAZ localized to the nucleus. In contrast, in MSCs cultured on soft substrates (0.7 kPa), YAP/TAZ remained in the cytoplasm, directing MSCs to undergo lipogenic differentiation (Dupont et al., 2011). MSC fate is also mediated through actinomyosin contractility and activated Rho/Rho kinase (ROCK) signaling (Mcbeath et al., 2004), as well as by mechanotransduction mediated by adhesion sites and integrins (Du et al., 2011). In response to increased stiffness, activated Rho stimulates actinomyosin stress fiber assembly, which in turn increases the cellular contractility and ERK activation, promoting osteogenic differentiation (Arnsdorf et al., 2009). Furthermore, Rho, in combination with the actin cytoskeleton, is essential for maintaining nuclear YAP/TAZ in MSCs. The activation of FAK via ROCK signaling resulted in upregulation of the osteogenic markers Runx2 and ALP and in matrix mineralization, indicating osteogenesis in human adipose stem/progenitor cells (Hyväri et al., 2018). In addition, the inhibition of FAK and ROCK signaling resulted in the upregulation of adipogenic markers. Finally, matrix stiffness regulates the osteogenic differentiation of MSCs through the Ras pathway, accompanied by increased Smad1/5/8, AKT and ERK phosphorylation. RasN17 inhibition leads to a significant decrease in Smad1/5/8, AKT and ERK activity and a significant decrease in osteogenic marker expression (Xue et al., 2013).

In conclusion, substrate stiffness emerges as a central regulator of MSC fate through a hierarchical mechanotransduction cascade: integrin-mediated focal adhesions transmit ECM rigidity to the actin–myosin cytoskeleton, generating contractile tension in stress fibers that is transduced via Rho/ROCK and FAK signaling into nuclear YAP/TAZ translocation. On soft matrices, cytoplasmic YAP/TAZ retention promotes adipogenesis; on rigid matrices, nuclear YAP/TAZ drives osteogenesis via Runx2, ALP, and matrix mineralization. These force-dependent pathways—amplified by adhesion density, RGD spacing, and actinomyosin contractility—highlight the need for biomaterials that precisely recapitulate tissue-specific stiffness and ligand presentation to direct stem cell differentiation.

## 3 Hydrogels

Hydrogels can be classified in numerous ways, for example, into natural and synthetic hydrogels according to their source. Natural hydrogel materials being investigated for use in tissue engineering include agarose, methylcellulose, hyaluronic acid, gelatin, chitosan, elastin-like polypeptides and other polymers of natural origin (El-Sherbiny and Yacoub, 2013). Common chemically synthesized components include polyacrylamide, polyvinyl alcohol, polyoxyethylene, polyacrylic acid and its derivatives, and other copolymers with abundant hydrophilic groups (Singh and Singhal, 2012).

**FIGURE 2:**
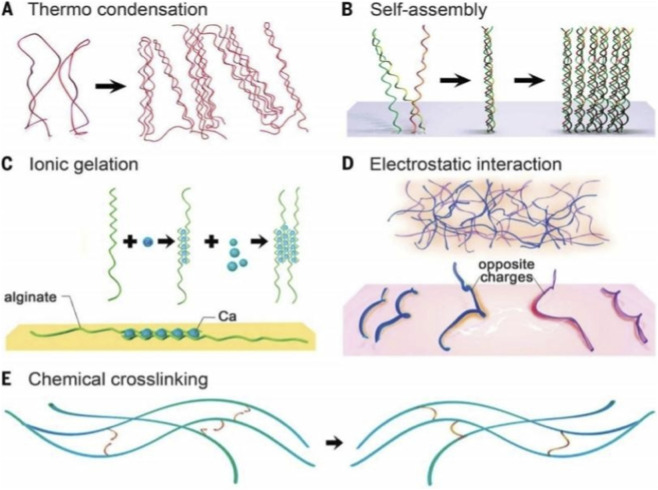
**(A)** Thermally induced entanglement of polymer chains. **(B)** Molecular self-assembly. **(C)** Ionic gelation. **(D)** Electrostatic interaction. **(E)** Chemical cross-linking. Reproduced with permission from Zhang and Khademhosseini (2017).

Hydrogels can form by physical entanglement, ionic interactions and chemical cross-linking. The majority of physical gelation methods depend on the intrinsic properties of the polymers. This dependence limits the ability to fine-tune the attributes of hydrogels, but gelation is easy to achieve without the need to modify the polymer chains and is usually easy to reverse when necessary. Conversely, chemical approaches can be used to allow more controllable, precise management of the cross-linking procedure, potentially in a spatially and dynamically defined manner. The physical cross-linking mechanisms include the thermally induced entanglement of polymer chains (Figure 2A), molecular self-assembly (Figure 2B), ionic gelation (Figure 2C) and electrostatic interactions (Figure 2D). Chemical cross-linking is better at stabilizing hydrogel matrices than physical methods because it allows for significantly greater flexibility and spatiotemporal precision during the gelation process than physical methods. Chemically active portions of the backbone or side chains of a macromolecule suspended in aqueous solution can form covalent bonds in appropriate cases to form hydrogels (Zhang and Khademhosseini, 2017) (Figure 2E).

Hydrogel properties, including water content, modulus and degradation rate, can be adjusted to match physiological conditions (Lee and Mooney, 2001). Hydrogels with tunable stiffness are important materials for studying the effect of matrix stiffness on cells. Covalent cross-linking strategies involve the formation of covalent linkages between the polymer chains of the hydrogel. The density of these covalent bonds limits how much the hydrogel will swell, which in turn determines the mechanical properties of the resulting hydrogel. Two main methods exist for fabricating substrates with varying stiffnesses. The first method is to adjust the cross-links in the hydrogel by varying the initial precursor concentration. The second method is to initiate the cross-linking of monomers, polymers or existing hydrogels and adjust the degree of reaction (Ting et al., 2021). Both reversible and irreversible changes in hydrogel networks can be induced using a variety of physical and chemical stimuli. Typical examples of physical stimuli include temperature and light, while chemical stimuli include pH, ions, voltage, and molecules or biomolecules that interact with the network (Tokarev and Minko, 2009).

Hydrogels are currently widely used in biomedical applications due to their excellent biocompatibility. Their functions include providing lubrication within the body (An et al., 2022), serving as drug carriers (Yi et al., 2022), and functioning as health monitoring sensors (Li et al., 2023). Moreover, hydrogels are good platforms for cell culture because of their great potential to mimic the natural ECM. Altering the various physical properties of hydrogels can facilitate studying the effects of mechanical cues on cell behavior. Hydrogels with fixed stiffness, while biocompatible and widely adopted, are inherently limited in recapitulating the dynamic mechanical heterogeneity of native ECM, which undergoes continuous remodeling. Such static substrates impose a single mechanical setpoint, constraining longitudinal studies of mechanosensitive processes—e.g., MSCs cultured on stiff (∼40 kPa) hydrogels exhibit irreversible osteogenic commitment after several weeks, precluding interrogation of stiffness-switching effects on lineage reversion (Lee et al., 2014) (Liu et al., 2025). In contrast, hydrogels with tunable stiffness enable on-demand, reversible modulation of elastic modulus (E) within physiologically relevant ranges without disrupting cell–matrix adhesions or 3D encapsulation (Li et al., 2025). These capabilities transform hydrogels from passive scaffolds into active, programmable microenvironments that faithfully emulate ECM dynamics, as summarized in Table 1.

**TABLE 1:** Summary of methods for tuning hydrogel stiffness.

Category	Mechanism	Representative method	Stimulus	Stiffness range	Cellular effect	References
Phototunable	Photo-crosslinking/Photodegradation	UV-induced crosslinking of HA/PEG, photoactive yellow protein (PYP) or o-nitrobenzyl-mediated degradation	UV (365 nm)/visible light	3 → 30 kPa 10 → 170 kPa 2.2 → 1.6 kPa	hMSC differentiation, mechanical memory and cellular traction forces	Khetan and Burdick (2010), Kloxin et al. (2009), Ondeck and Engler (2016), Brown et al. (2018), Rosales et al. (2017), Yang et al. (2025)
	Photo-conformational switching	Azobenzene cis–trans isomerization	UV (365 nm) ↔ visible light (400–500 nm)	2 → 8 kPa	Cell morphology, YAP localization	Rosales et al. (2015), Lee et al. (2018)
	Photo-host–guest interaction	CB[8]/β-CD photodimerization/uncoupling	240 nm (softening) ↔ 320–390 nm (hardening)	78 → 1,000 Pa	Fibroblast viability	Anthony et al. (2019), Rosales et al. (2018)
Chemo-/Enzymatic	Enzymatic hydrolysis	MMP/collagenase-degradable peptide cleavage	Collagenase	0.1 → 42 kPa	Fibroblast migration, spreading	Qin et al. (2018)
	Chemical ion release	Slow Ca^2+^ release (CaCO_3_ + GDL)	Chemical	Not specified	rMSC differentiation	Chen and Lv (2022)
Ionic/Electro	Ionic crosslinking	Ca^2+^-alginate or voltage-controlled ion binding	NIR, electrical conditioning	151 → 1,074 Pa 10 → 30 kPa	Stiffness gradients	Stowers et al. (2017), Yang and Liang (2018)
Redox/Conformational	Disulfide bond folding/unfolding	Oxidation/reduction	Oxidant/reductant	40 → 10 kPa	Lung fibroblast morphology	Kong and Li (2016), Fu et al. (2019)
Nucleic/H-bond	DNA complementary crosslinking	Addition/removal of complementary strands	DNA strand exchange	194 → 691 Pa 5.8 → 22.9 kPa	Neurite outgrowth, cell polarity	Lin et al. (2004), Jiang et al. (2010a), Jiang et al. (2010b)
	pH-responsive i-motif	DNA i-motif contraction/extension	pH 5.0–8.0	35 → 1,000 Pa	Fully reversible	Deshpande et al. (2017), Zhou et al. (2016)

↔ denotes fully reversible modulation; single arrows indicate irreversible changes. Stiffness values are reported as initial → final (or range).

### 3.1 Irreversible stiffness modulation

Most irreversible changes in hardness are made by chemical reactions that alter the cross-linked network. This includes methods such as hydrolyzing the chemical bonds of the hydrogel, leading to softening and the formation of new cross-links to harden the hydrogel. Several studies have shown that the hardness of hydrogels can be altered unidirectionally by changing the physical cross-linking density.

As a noninvasive stimulus, UV light can harden preformed hydrogels by inducing additional cross-links. Guvendiren and Burdick’s study revealed a specific type of hyaluronic acid hydrogel characterized by its cross-linking reaction using dithiothreitol triggered by UV irradiation with the Irgacure 2,959 photoinitiator. When hMSCs were grown on a very soft hydrogel (3 kPa), they exhibited adipogenic properties, whereas on a rigid substrate (30 kPa), they predominantly exhibited bone-forming properties. In addition, the hardening time of the substrate and the differentiation status of the cells at the time of hardening have a crucial influence on determining the response of the cells (Khetan and Burdick, 2010). UV light can be used not only to harden hydrogels but also to hydrolyze or soften them. Anseth’s group prepared a tunable photodegradable hydrogel with PEG diacrylate and o-nitrobenzyl (Kloxin et al., 2009) to study fibrillization and the “mechanical memory” (Yang et al., 2014) of hMSCs.

Ondeck and Engler invented a hydrogel based on methacrylate hyaluronic acid (MeHA), which is notable for the tuning of its mechanical properties by sequential photopolymerization. This hydrogel was formed by photopolymerization with Irgacure 2,959, which is characterized by cross-linked methacrylate groups. The amount of cross-linking in the hydrogel can be controlled by adjusting the exposure time. The stiffness of the MeHA matrix increases from 10 to 70 kPa after an exposure time of 1 min, to 30–170 kPa after the material is subsequently cross-linked for an additional 1 min (Ondeck and Engler, 2016). In addition, Anseth’s team created an azobenzene-PEG hydrogel containing dioxolane groups, which hardened upon exposure to 365 nm light. By adjusting the ratio of alkene to azide, the initial storage modulus of 3.7 kPa could be increased to 8.2 kPa, and both the initial modulus and modulus changes were modulated. After hardening, the encapsulated C2C12 mouse myoblasts exhibited reduced the proliferation and the nuclear localization of Yes-associated protein (YAP), indicating that myoblasts are also sensitive to dynamic changes in matrix stiffness (Brown et al., 2018).

The modulation of the mechanical properties of most photosensitive hydrogels is unidirectional and mainly involves simply increasing or decreasing the modulus. To simulate the dynamic characteristics of natural ECM, Anseth and colleagues modified HA with both methyl acrylate and o-nitrobenzyl acrylate (Rosales et al., 2017). The acrylate group is more reactive due to its higher electrophilicity; therefore, added dithiothreitol (DTT) preferentially reacts with the o-nitrobenzyl acrylate group through thiol-ene addition to form a photocleavable cross-link. UV radiation can degrade o-nitrobenzyl acrylate cross-links and soften hydrogels without affecting the methyl acrylate groups in the absence of a photoinitiator. Therefore, methyl acrylate groups can be used to subsequently harden hydrogels by exposing them to 400–500 nm light in the presence of a photoinitiator. Using this “soft-hard-soft” hydrogel, the authors found that reducing or increasing the stiffness of the hydrogels led to a decrease or increase in the hMSC spreading area, respectively. These results clearly demonstrated that cells can sense and respond to dynamic changes in hydrogel mechanical properties. The cross-linked structure of hydrogels can also be altered by enzymatic hydrolysis. In a broad sense, all hydrogels composed of enzymatically degraded natural polymers (e.g., HA, collagen, gelatin) can be degraded by catabolic enzymes (e.g., hyaluronidase and metalloproteinases) to alter the network dynamics (Park et al., 2003).

Qin et al. developed photopatterned hydrogels containing MMP-degradable peptides (Qin et al., 2018). Analysis of human dermal fibroblasts encapsulated in this system confirmed that cell viability, spreading, and migration in the hydrogels were affected by the photopatterned adhesion ligands and cell-mediated proteolytic hydrolysis.

**FIGURE 3:**
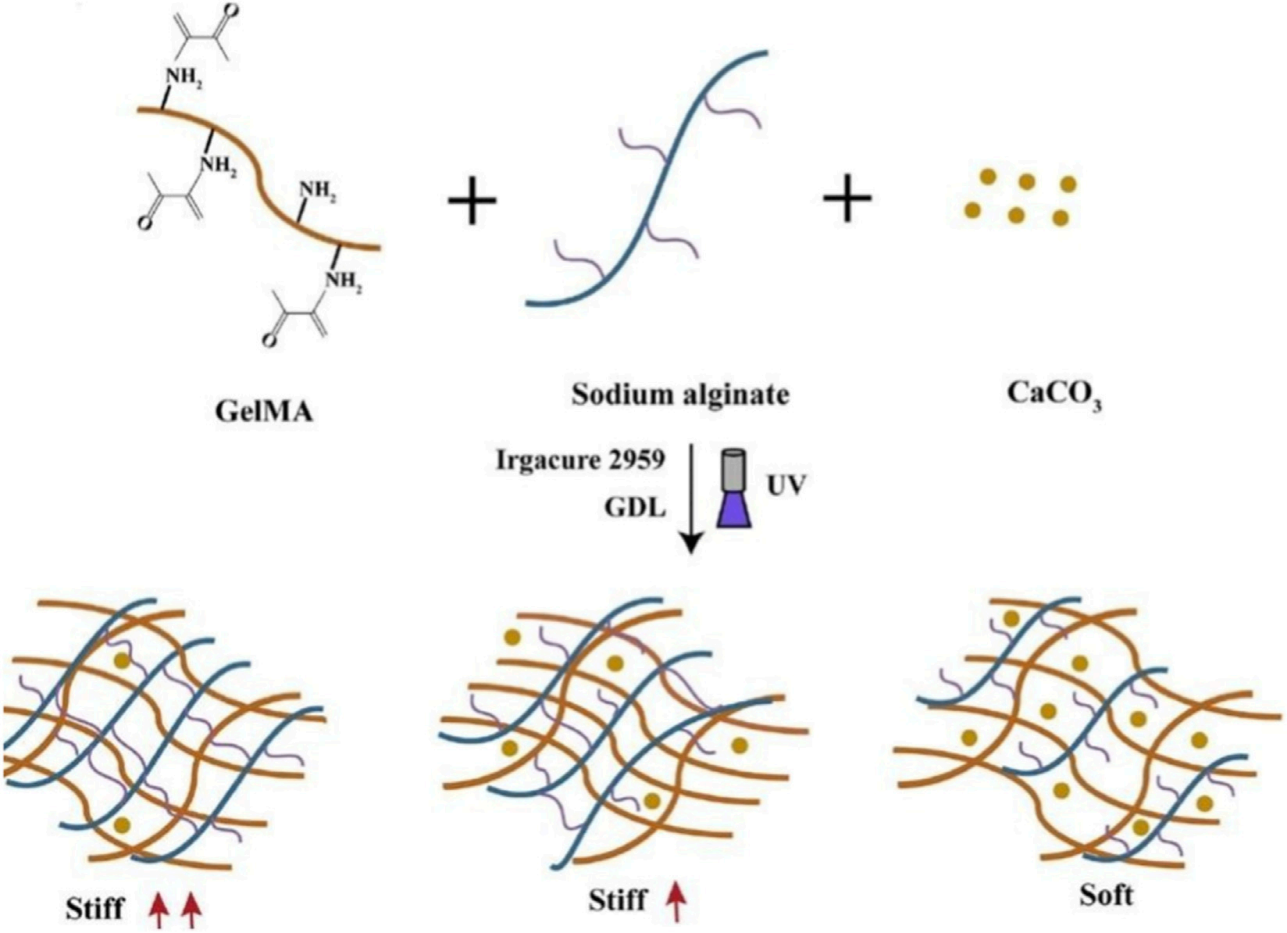
Synthesis of GelMA/SA hydrogels with dynamic stiffness. Reproduced with permission from Qin et al. (2018).

Similarly, varying the density of a physically cross-linked network can regulate the strength of the hydrogel. Physical cross-links can be formed through interactions between the gulo-glucuronide region of alginate and calcium ions. The release of calcium or a calcium chelator was controlled by near-infrared (NIR) light-triggered release from temperature-sensitive liposomes containing gold nanorods to induce localized heating. In this system, the unirradiated control gel had an elastic modulus of 151 Pa, similar to that of normal breast tissue, and after laser irradiation, the gel hardened to 1,074 Pa (Stowers et al., 2017). Lv’s team developed a gelatin methacryloyl/sodium alginate (GelMA/SA) composite hydrogel with CaCO3 and GDL encapsulated to control the release of calcium ions, leading to slow cross-linking with SA, which resulted in a slow and dynamic increase in matrix stiffness. The dynamically hardened hydrogel significantly affected the diffusion, adhesion and osteogenic differentiation of rMSCs (Chen and Lv, 2022) (Figure 3).

Research on irreversible changes in hydrogel stiffness has helped us to better understand the effects of matrix stiffness on cell behavior. The timing of hardening/softening has also been found to affect MSC differentiation (Yang et al., 2014). To better understand the effects of dynamic mechanical properties, a gradually increasing number of studies on reversibly hardening/softening hydrogels are being performed to simulate the dynamic remodeling of the ECM more closely.

### 3.2 Reversible stiffness modulation

Hydrogels with reversible stiffness changes are typically formed through reversible interactions between polymer chains, including dynamic chemical cross-linking and physical cross-linking. In general, physical cross-links, such as host-guest interactions, ionic interactions, and hydrogen bonding, are characterized as transient interactions, which can lead to greater dynamics and more unstable networks than those in hydrogels formed via chemical/covalent cross-links. In this section, several fabrication principles and examples of hydrogels with reversible stiffening are briefly described.

#### 3.2.1 Dynamic hydrogels cross-linked by conformation-changeable linkers

Certain hydrogel structural units, including those containing small-molecule cross-linkers or proteins, can undergo significant conformational changes in response to specific stimuli (e.g., light or ligand interactions) (Ehrick et al., 2005). These conformational changes can manifest in a variety of ways that alter the structure and corresponding dynamic properties of the hydrogel network.

Azobenzene and its derivatives exhibit unique photogenic trans-cis isomerization behavior. In small-molecule systems, the irradiation of azobenzene with 360 nm light induces a conformational transition from the stable trans configuration to the unstable cis configuration; however, removal of the light source or irradiation in the visible range (400–500 nm) results in almost complete recovery of the trans isomer (Zhao and Stoddart, 2009).

In 2015, the Anseth team developed a PEG-based hydrogel featuring azobenzene for reversible light-induced cross-linking (Rosales et al., 2015). In these hydrogels, the peptide cross-linkers softened under 365 nm UV irradiation (trans-to-cis isomerization) and hardened under 400–500 nm visible light, with photoisomerization disrupting hydrogen bonding and reducing the storage modulus. The isomerization of azobenzene is reversible. Valve mesenchymal cells encapsulated in the gel maintained high levels of viability and achieved diffuse fibroblast morphology, with some of the cells expressing a myofibroblast phenotype.

**FIGURE 4:**
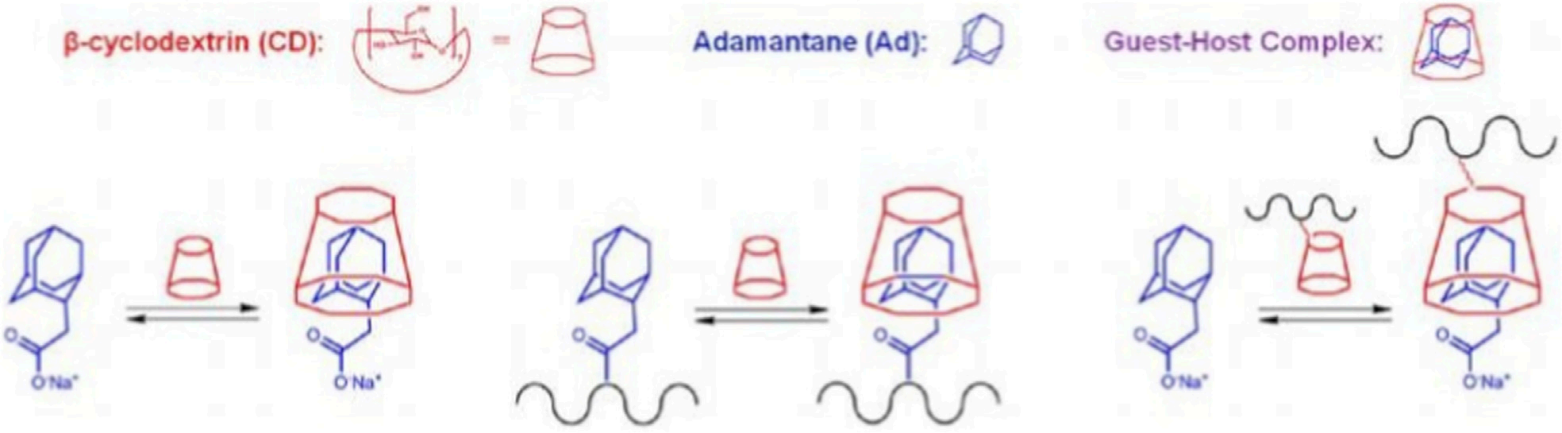
Confirmation of guest−host complexation for modified macromers by titration of the guest (adamantane, Ad) by the host (β-cyclodextrin, CD). Schematic of the Ad and CD interactions examined, either alone or when either Ad or CD is coupled to HA (illustrated as black line). Reproduced with permission from Rodell et al. (2013).

Scientists have developed several hydrogels using azobenzene to study the effects of reversible stiffness changes on cells. PAAm hydrogels containing azobenzene exhibited reversible changes in Young’s modulus, which decreased from 8.3 ± 2.0 kPa (trans state) to 2.0 ± 0.6 kPa (cis state) after 365 nm irradiation. The authors reported that stiffening resulted in increased extension and higher aspect ratios of bone marrow-derived MSCs (Lee et al., 2018) (Figure 4).

Disulfide bonds and thioesters are common in living organisms and play crucial roles in biosynthesis. Under physiological conditions, these bonds can be reversibly formed and broken in the presence of oxidizing/reducing agents, contributing to dynamic conformational changes in natural proteins (Xu et al., 2016). Kong et al. fabricated redox-responsive hydrogels using protein cross-linkers (Kong and Li, 2016). Through the oxidation and reduction of disulfide bonds, proteins can switch between folded and unfolded conformation states, leading to reversible changes in hydrogel physical properties. The oxidized state corresponds to the shorter chain length that results from the folded conformation between cross-linking sites, resulting in a decreased solubility at 40 kPa. In contrast, the reduced state has a longer chain length, which leads to a softer, more hydrated structure (10 kPa). In later work, they demonstrated that human lung fibroblasts dynamically and reversibly change their morphology when Young’s modulus is cyclically varied between 20 kPa and 6 kPa (Fu et al., 2019). However, redox compounds may affect other cellular behaviors and need to be thoroughly studied.

#### 3.2.2 Dynamic hydrogels cross-linked by ionic interactions

Electrostatic interactions are common in biocomplexes and can be used in effective strategies for generating ionic complexes, mediating biomolecular attachment and stabilizing molecular conformations to prolong biomolecular activity. Such strategies have recently received increasing attention in the construction of dynamic hydrogels for various biomedical applications (Gao et al., 2015).

For example, alginate, a naturally occurring anionic polymer derived from kelp, has been used in a wide range of biomedical applications, including wound healing, tissue engineering and drug/protein delivery (Canavan et al., 2005). The structure of the alginate molecule allows strong ionic interactions and a high degree of coordination with divalent or trivalent ions, resulting in the formation of ionically cross-linked dynamic hydrogels. Unlike covalent cross-linking, alginate cross-linking is reversible, and the cross-linking density can be reduced to soften 3D block gels. By varying the voltage and duration, any stiffness between 10 kPa and 30 kPa can be obtained. Although a wide range of adjustable stiffnesses is available, the stiffness is not strictly controllable. Experiments showed that the electrical conditioning method allows ionic cross-linking to dynamically manipulate the material properties of the hydrogel to generate a dynamic gradient, the value of which can be adjusted by electric charge. As the charge increases, the stiffness of the hydrogel near the anode increases, while the stiffness of the hydrogel near the cathode decreases (Yang and Liang, 2018).

#### 3.2.3 Dynamic hydrogels cross-linked by host–guest interactions

As a subset of supramolecular chemistry, host‒guest interactions show great potential for the fabrication of dynamic hydrogels (Mantooth et al., 2019). Host‒guest interactions are based on the transient association of a molecule containing a hole (i.e., the host) with a suitable molecular guest. Typically, either naturally sourced (e.g., cyclodextrins) and synthetic (e.g., cup[n]arnes, etc.) macrocycles can serve as host molecules. The criteria for host–guest pairs include the complementary sizes of the host cavity and the guest molecule as well as their specific interactions, such as hydrophobic interactions, hydrogen bonding, and charge–dipole interactions.

Cucurbit[n]ureas (n = 5–8, 10; CBs) are a family of macrocyclic compounds self-assembled by an acid-catalyzed condensation reaction between glyburide and formaldehyde (Freeman et al., 1981). CBs have hydrophobic cavities and a symmetric “barrel” shape with two identical gate regions connected by a urea-carbonyl oxygen. The number of glycoluronium units determines the size of the mengluronium cavity so that smaller homologs of the CB[n] family (i.e., n = 5–7) can bind a single guest, whereas CB[8] can accommodate two guests simultaneously. The equilibrium of the binding between CB[n] and the corresponding guest molecules provides the basis for the use of CB to produce transient cross-linking.

Supramolecular coumarin-functionalized hydrogels formed by host-guest-mediated self-assembly with cucurbit[8]uril (CB[8]) also exhibit reversible changes in mechanical properties (Anthony et al., 2019). CB[8] is a photosensitive molecule that undergoes [2 + 2] photodimerization at 4,310 nm and reversibly uncouples at 310 nm (Trenor et al., 2004). This property allows supramolecular hydrogels to form noncovalent cross-linking when stimulated by transformation to covalent cross-linking. The authors prepared hyaluronic acid hydrogels whose stiffness could be reversibly adjusted from 78 Pa to 360 Pa under 240 nm (softening) and 320–390 nm (hardening) irradiation. Despite this reversibility, the wavelengths responsible for stimulating CB[8] are incompatible with cells and tissues, which limits its biological application.

**FIGURE 5:**
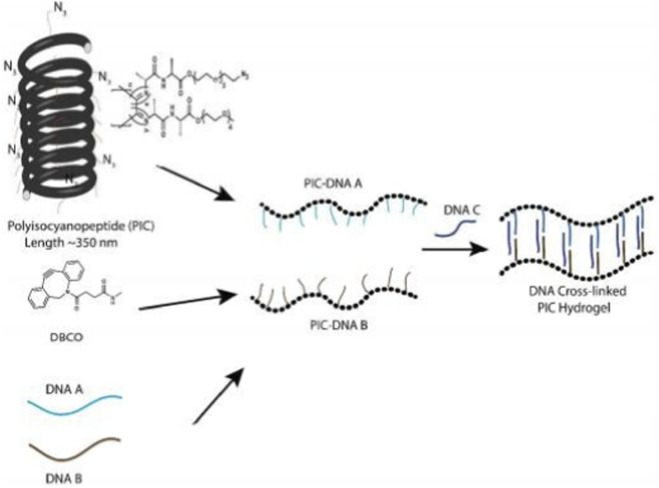
Preparation of DNA-responsive PIC hydrogels. The azide-functionalized PIC polymer is conjugated with ssDNA using DBCO and strain-promoted click chemistry. Two separate batches of PIC polymers conjugated with DNA A or PIC-DNA B are mixed together with complementary cross-linker DNAs to form the hydrogel. Reproduced with permission from Deshpande et al. (2017).

Cyclodextrins (CDs) are the most ubiquitous group of subject macrocyclic compounds because of their high-water solubility, low toxicity, and wide range of uses (Szejtli, 1998). Based on the number of dehydrated glucose (D-glucose) units, CDs containing six, seven, or eight D-glucose repeating units were named α-, β-, or γ-CDs, respectively. The hydroxyl groups of CDs are located on the solvent-exposed outer surface, thus forming a hydrophobic inner cavity that can accommodate hydrophobic guest molecules (Davis and Brewster, 2004) (Figure 5). These molecular interactions between CDs and their corresponding guests have been used to form supramolecular hydrogels (Liu et al., 2018).

The host and guest motifs can also be grafted onto the polymer backbone prior to the gelation process. Burdick and colleagues first grafted β-CD and Ada to the HA backbone and subsequently fabricated the host-guest hydrogel by simply mixing the two polymer solutions (Rodell et al., 2013). Burdick et al. prepared hyaluronic acid hydrogels with reversible stiffness using azobenzene and β-cyclodextrin. The stiffness was altered by isomerization with 365 nm UV or 400–500 nm visible light to change the binding affinity between azobenzene and β-cyclodextrin, leading to changes in network connectivity. The hydrogel modulus was reversible between 600 Pa and 1,000 Pa, where softening was achieved at 365 nm and hardening was restored at 420 nm. Encapsulated NIH 3T3 fibroblasts maintained a high level of cell viability (90%) after 3 days in culture (Rosales et al., 2018).

#### 3.2.4 Dynamic hydrogels cross-linked by hydrogen bonding

Dynamic hydrogels can also be formed by utilizing hydrogen bonding interactions (Zhao et al., 2016). Inspired by hydrogen bonding between complementary nucleotide pairs, Tan et al. reported the fabrication of self-assembling hydrogels by mixing four-armed PEGs concatenated with adenine and thymine (Tan et al., 2012). This hydrogel is injectable and exhibits excellent cell encapsulation and growth factor delivery ability. Langrana’s group first fabricated a PAAm hydrogel with regions binding a DNA strand to support secondary DNA cross-linking (Lin et al., 2004). The interconnectivity was increased by the addition of a DNA strand with a complementary base sequence. Cross-linking was reversed through competitive binding by the addition of complementary removal strands, which eliminated the secondary cross-linking in the hydrogel. A small change in stiffness from a minimum of 194 Pa to a maximum of 691 Pa was observed upon DNA strand addition. The migration of the strands was accelerated using electrophoresis. In later work, Langrana and coworkers copolymerized PAAm with DNA strands covalently attached to Acrydite™ polymer chains, and cross-linking was induced by L2 DNA strands (Jiang et al., 2010a). Gelation was reversed when the complementary L2 strand was introduced. The stiffness could be tuned between 5.8 and 22.9 kPa by adjusting the number of DNA strands added. L929 and GFP fibroblasts exhibited different projection areas and polarities, depending on the cell line, in response to dynamic stiffness. The authors subsequently applied their system to investigate neurite outgrowth (Jiang et al., 2010b).

Dynamically adjustable DNA hydrogels with mechanical properties regulated by pH can be fabricated using semiflexible polyisocyanatopeptide polymers and DNA cross-linkers (Deshpande et al., 2017). Hydrogels fabricated at pH 7.4 exhibit an energy storage modulus of 35 Pa, and their elastic modulus can be changed to 100 Pa by adjusting the pH to 5.2. This hardening is attributed to the contraction of the DNA i-motif sequences, which results in a stiffer DNA cross-linker. The hardening was shown to be fully reversible by returning the pH to 7.4.

DNA-based pH-responsive hydrogels have been fabricated using a Y-shaped scaffold formed by three single-stranded DNAs with “sticky” ends (Zhou et al., 2016). The ends are linked together by a linear pH-responsive DNA i-matrix junction. The i-matrix changes from a four-stranded structure to a single-stranded structure when the ambient pH exceeds 6.3, which allows the reversible adjustment of the mechanical energy storage modulus between 250 Pa (pH 8.0) and 1,000 Pa (pH 5.0).

## 4 Biomedical applications of hydrogels with tunable stiffness

Hydrogels with tunable stiffness are versatile biomaterials that have garnered significant attention in biomedical research due to their ability to mimic the dynamic mechanical properties of the ECM. By precisely controlling their mechanical properties, these hydrogels provide a platform to investigate how mechanical cues influence cellular behavior and enable advanced applications in regenerative medicine, immunotherapy, and organoid culture. This chapter reviews recent advancements in these areas, highlighting specific examples of hydrogels with tunable stiffness and their impact on biomedical applications. A summary table (Table 2) is provided to categorize these applications, detailing the hydrogel type, stiffness range, biological effects, and corresponding references.

**TABLE 2:** Biomedical applications of hydrogels with tunable stiffness.

Application	Representative hydrogel	Stiffness range	Biological outcome	References
Stem cells and regenerative medicine	Photodegradable PEG-diacrylate/HA-MMP/Ada-grafted HA-CD	3–30 kPa (or not specified)	Temporal softening → osteogenic; rigid or non-degradable → adipogenic; enhanced *in vivo* cartilage/collagen	Khetan and Burdick (2010), Yang et al. (2014), Wei et al. (2016)
Organoid culture	sPEG-dPEG/PEG/GelMA/synthetic dynamic	0.3–10 kPa	Rigid → ISC expansion; intermediate → intestinal growth; soft → hepatic impairment; high → kidney nephron structures	Gjorevski et al. (2016), Sorrentino et al. (2020), Clerkin et al. (2025), Rijns et al. (2025)
Immunoregulation and immunotherapy	HA with T-cell ligands/alginate methacrylate (ALMA)	0.25–4.5 kPa	Optimized stiffness + ligands → robust T-cell expansion and tumor inhibition; macrophage polarization regulation	Hickey et al. (2019), Coser et al. (2025)
Wound healing	Photopatterned PVA-MMP/poly(amidoamine) (PAA)-based poly(n-isopropyl acrylamide) (PNIPAM)	0.1–42 kPa	Gradient stiffness → enhanced fibroblast migration and *in vivo* repair; myofibroblast transformation, keratinocytes proliferation, extracellular matrix synthesis and remodeling	Qin et al. (2018), Chen et al. (2016)
Drug delivery and cartilage repair	Kartogenin-composite/GelMA	5–20 kPa 7–45 kPa	Sustained release + improved chondrogenesis mRNA delivery	Li et al. (2023), Athirasala et al. (2022)

### 4.1 Stem cells and regenerative medicine

Because of their capacity for self-renewal and their ability to differentiate into various cell types in the body, stem cells have great potential as novel therapeutic agents for regenerating or replacing functionally impaired tissues. Numerous studies involving different types of stem cells have been conducted, and initial clinical success has been reported (Liu et al., 2020). In many cases, *in vitro* expansion of stem cells prior to transplantation is necessary to achieve satisfactory cell numbers. In addition, the survival, proliferation and specific differentiation of transplanted cells are important for achieving their intended therapeutic function. Hydrogels with dynamic stiffness can control stem cell fate in two major ways: maintenance of stemness and differentiation into the desired mature cell lineage.

As previously described, Yang et al. used hydrogels that can be dynamically softened by UV-triggered degradation; these hydrogels have been further used to study the temporal effects of substrate stiffness and adaptation on MSC differentiation (Yang et al., 2014). MSCs cultured on the softened stiff gel on day 1 postinoculation retained the ability to differentiate into osteoblasts and adipocytes. In contrast, MSCs cultured on softened hard gels after 7 days were committed only to osteogenic differentiation. Furthermore, Khetan et al. encapsulated MSCs in HA hydrogels cross-linked with MMP-responsive peptides and demonstrated that hydrogel degradation-induced network dynamics facilitated cell spreading in a 3D environment and promoted osteogenic differentiation (Khetan and Burdick, 2010). In contrast, cells encapsulated in nondegradable hydrogels exhibited mainly adipogenesis. Subsequently, the authors introduced secondary static cross-linking into the hydrogels, thereby eliminating the network of dynamic properties. This led to a shift from osteogenesis-dominant to adipogenesis-dominant differentiation, highlighting the role of dynamic hydrogels in controlling MSC differentiation.

Wei et al. developed two-component host–guest hydrogels assembled from Ada-grafted HA and *in situ* photopolymerized acryloyl-β-CD. Compared to covalently cross-linked MeHA hydrogels, these hydrogels exhibit enhanced mechanical properties while retaining the dynamic features necessary to enable encapsulated human MSCs to form cartilage with increased collagen deposition and to promote cartilage regeneration *in vivo* (Wei et al., 2016). These studies emphasize the ability of tunable stiffness hydrogels to direct stem cell differentiation for applications such as bone and cartilage regeneration.

### 4.2 Immunoregulation and immunotherapy

The immune system is a delicate and dynamic network that defends the organism against pathogens and maintains homeostasis. Immune cells sense and respond to biochemical and biomechanical cues from neighboring cells or from the surrounding ECM during their development, activation, differentiation, or effector functions (Liu and Segura, 2020). There is increasing evidence that biomechanical signals from the ECM play a key role in determining immune cell function and regulation.

Hydrogels with dynamic stiffness can increase immune cell efficacy. Autologous adoptive T-cell transfer is a technique that involves extracting T cells from patients, followed by engineering, expanding, and reintroducing these *ex vivo* generated autologous T cells (Liu and Segura, 2020). Although significant progress has been made in the development of this therapy, many challenges remain to be overcome. For example, transfusion typically requires large numbers of therapeutic T cells (typically 10^10^ cells or more), and the process is time-consuming and relatively expensive. Hickey et al. recently designed a 2D HA-based hydrogel matrix in which the density of T-cell stimulating ligands (anti-CD3 and anti-CD28), substrate hardness, and cell-adhesion ligand density can be independently controlled (Hickey et al., 2019). They found that optimization of these biochemical and biophysical properties of the engineered matrices led to the rapid and robust expansion of functional T cells *in vitro*; further overtransfer of these cells strongly inhibited tumor growth in mice.

### 4.3 Organoids

One of the key advances in the field of stem cell research in the past decade has been the development of organoids generated from self-renewing stem cells, including pluripotent stem cells and tissue-derived stem cells (Clevers, 2016). Organoids are unique 3D cellular models with multicellular structures that resemble organs and can replicate some of the functions of the corresponding tissues, such as the intestine, liver, and brain (Sorrentino et al., 2020). Due to their unique characteristics, organoids offer unprecedented opportunities for basic research on organ development and disease pathogenesis, and they are powerful tools for drug screening and reliable sources for future organ transplantation. Hydrogels with tunable stiffness are critical for recapitulating the dynamic mechanical environment of native tissues, which influences organoid formation and maturation.

Lutolf and colleagues performed 3D culture of intestinal stem cells (ISCs) in synthetic hybrid sPEG-dPEG hydrogels with tunable degradability and rigidity for the derivation of intestinal organoids. This study demonstrated that an initially rigid hydrogel matrix (1.3 kPa) is required for ISC expansion and that degradation-induced softening of the hydrogel matrix (200 Pa) is critical for subsequent organoid morphogenesis. These findings emphasize the importance of mimicking the dynamic evolution of natural ECM structural properties in designed hydrogels to facilitate organoid development in culture (Gjorevski et al., 2016). Similarly, Sorrentino et al. used PEG-based hydrogels with tunable stiffness (0.3–4 kPa) to culture liver organoids, showing that harder matrices (4 kPa) exhibited significant impairment of hepatic differentiation and organoid functionality, as evidenced by a reduction in the expression of hepatic progenitor markers (Sorrentino et al., 2020).

Clerkin et al. further advanced organoid culture by developing a semi-synthetic GelMA hydrogel with dynamic stiffness (400–10,000 Pa) for kidney organoid derivation (Clerkin et al., 2025). These hydrogels supported nephron-like structure formation after their stiffness was optimized to match embryonic kidney tissue, demonstrating their potential for renal tissue engineering. Another study by Cruz-Acuña et al. utilized synthetic dynamic hydrogels with tunable stiffness to culture intestinal organoids, showing that intermediate stiffness (0.7 kPa) promoted mechanical support and organoid growth. These studies underscore the importance of dynamic stiffness in recapitulating tissue-specific mechanical cues for organoid development (Rijns et al., 2025).

### 4.4 Other biomedical applications

Beyond stem cells, immunotherapy, and organoids, hydrogels with tunable stiffness have applications in other biomedical fields. For instance, Qin et al. developed photopatterned PVA hydrogels (0.1–42 kPa) with MMP-degradable peptides for wound healing, demonstrating enhanced fibroblast migration and tissue repair *in vivo* (Qin et al., 2018). In drug delivery, Li et al. created kartogenin-conjugated composite hydrogels with tunable stiffness (5–20 kPa) for cartilage repair, achieving sustained drug release and improved chondrocyte differentiation (Li et al., 2023). These examples highlight the versatility of tunable stiffness hydrogels in addressing diverse biomedical challenges.

## 5 Summary and outlook

The ECM functions as a temporally adaptive mechanical scaffold whose stiffness evolves in concert with physiological demands, exerting hierarchical control over cellular behavior through force-dependent signaling cascades (Geiger et al., 2009). This dynamic rigidity, ranging from sub-kPa compliance in neural microenvironments to tens of kPa in musculoskeletal tissues, is transduced via integrin-mediated focal adhesions, actomyosin contractility, and nucleocytoplasmic shuttling of mechanosensitive effectors such as YAP/TAZ, ultimately imprinting epigenetic states that govern lineage specification, tissue remodeling, and pathological progression (Kayal et al., 2020; Padhi and Nain, 2020). Hydrogels with programmable stiffness have evolved into indispensable platforms for interrogating these mechanotransductive principles, enabling precise spatiotemporal manipulation of elastic modulus within physiologically relevant regimes.

This review establishes a comprehensive ontological framework that unifies irreversible stiffness modulation—typically mediated by covalent bond formation, photodegradation, or enzymatic network remodeling—with the complete repertoire of reversible crosslinking paradigms, including conformationally switchable linkers, ionic coordination equilibria, supramolecular host–guest inclusion, and dynamic hydrogen-bonding assemblies. This classification transcends modality-specific analyses by positioning all mechanisms within a shared continuum of force transmission kinetics, thereby revealing their convergent roles in recapitulating ECM temporal heterogeneity.

### 5.1 Emergent mechanobiological principles

A unifying insight across diverse systems is the primacy of stiffness transition dynamics over static elastic modulus in dictating cellular response (Atcha et al., 2021). The velocity of rigidity change, directional hysteresis during cyclic modulation, and reversibility of mechanical states collectively dominate cytoskeletal tensegrity, nuclear envelope mechanics, and the persistence of mechanomemory (Wang et al., 2025). These parameters determine the threshold for sustained YAP/TAZ nuclear residency, the maturation of stress fiber architectures, and the epigenetic encoding of prior mechanical experience, establishing rate-dependent setpoints that override equilibrium stiffness in lineage commitment and tissue patterning.

### 5.2 Temporal kinetics of mechanotransduction

Force propagation kinetics, rather than steady-state rigidity, emerge as the dominant instructive signal in cellular decision-making. Rapid stiffening induces transient hypercontractility and irreversible osteogenic priming through prolonged nuclear YAP retention, whereas controlled softening dissipates cytoskeletal tension and enables lineage reversion via cytoplasmic sequestration of mechanotransducers (Thomasy et al., 2013). Stress relaxation kinetics, independent of elastic modulus, further modulate cell spreading and differentiation thresholds, with fast-relaxing networks favoring chondrogenic phenotypes and slow-relaxing matrices promoting fibrotic transdifferentiation. These principles, validated across multiple length scales, underscore the necessity of programmable transition rates to faithfully emulate native ECM remodeling trajectories (Chaudhuri et al., 2016).

### 5.3 Translational challenges and strategic roadmap

Clinical translation of stiffness-tunable hydrogels is constrained by four interdependent barriers that demand integrated chemical, biological, and engineering solutions. First, *in vivo* mechanical fidelity is compromised by nonspecific degradation in proteolytic and oxidative microenvironments, resulting in premature loss of programmed stiffness within 1–2 weeks (Holloway et al., 2014). Orthogonally tunable dynamic-covalent networks combining hydrazone exchange with thioester equilibria offer extended functional lifetimes while maintaining reversibility, but require rigorous pharmacokinetic modeling to ensure byproduct biocompatibility (Chen et al., 2023). Second, host–matrix feedback dyscrasia arises from maladaptive mechanotransductive loops, wherein excessive stiffening sustains pathological YAP/TAZ signaling and myofibroblast activation, while abrupt softening triggers anoikis in anchorage-dependent lineages. Patient-specific mechanical profiling via elastography, coupled with adaptive modulus trajectories, is essential to maintain physiological setpoints and prevent fibrotic encapsulation (Sharip and Kunz, 2025). Third, vascular integration remains limited by spatiotemporal mismatches between stiffness gradients and endothelial sprouting dynamics. Viscoelastic dissonance disrupts tubulogenesis beyond 14 days, necessitating bioinstructive gradient engineering through strain-promoted cycloadditions to synchronize angiogenic cue presentation with mechanical evolution (Wei et al., 2020). Fourth, regulatory and manufacturing harmonization is complicated by the classification of dynamic systems as combination products, requiring dual demonstration of device reliability and biologic safety. Cytotoxic photoinitiators and batch-to-batch variability in crosslinking density impede GMP scalability, mandating transition to visible-light-mediated polymerization and closed-loop automation platforms.

### 5.4 Outlook

The convergence of materials chemistry, systems biology, and regulatory science will drive the development of autonomous 4D hydrogels capable of closed-loop mechanical homeostasis. Integration of embedded microsensors with genetically encoded actuators will enable real-time stiffness adaptation in response to tissue strain, inflammatory markers, or metabolic flux. Machine learning frameworks trained on high-dimensional mechanotransduction datasets will accelerate the inverse design of chemistry–biology interfaces with prescribed temporal profiles. Under the Food and Drug Administration (FDA)’s 2025 Regenerative Medicine Advanced Therapy (RMAT) designation, over 50 requests are granted, targeting musculoskeletal, neural, and vascular regeneration. Success hinges on sustained interdisciplinary collaboration to transform stiffness-tunable hydrogels from passive scaffolds into active orchestrators of tissue destiny, redefining the interface between synthetic materials and living systems.
